# Detection of Prokaryotic Genes in the *Amphimedon queenslandica* Genome

**DOI:** 10.1371/journal.pone.0151092

**Published:** 2016-03-09

**Authors:** Cecilia Conaco, Pantelis Tsoulfas, Onur Sakarya, Amanda Dolan, John Werren, Kenneth S. Kosik

**Affiliations:** 1 Marine Science Institute, University of the Philippines, Diliman, Quezon City, Philippines; 2 University of Miami School of Medicine, Departments of Neurosurgery and Cell Biology, Miami, Florida, United States of America; 3 Natera, San Carlos, California, United States of America; 4 Biology Department, University of Rochester, New York, United States of America; 5 Neuroscience Research Institute and Department of Molecular Cellular and Developmental Biology, University of California, Santa Barbara, United States of America; University of New South Wales, AUSTRALIA

## Abstract

Horizontal gene transfer (HGT) is common between prokaryotes and phagotrophic eukaryotes. In metazoans, the scale and significance of HGT remains largely unexplored but is usually linked to a close association with parasites and endosymbionts. Marine sponges (Porifera), which host many microorganisms in their tissues and lack an isolated germ line, are potential carriers of genes transferred from prokaryotes. In this study, we identified a number of potential horizontally transferred genes within the genome of the sponge, *Amphimedon queenslandica*. We further identified homologs of some of these genes in other sponges. The transferred genes, most of which possess catalytic activity for carbohydrate or protein metabolism, have assimilated host genome characteristics and are actively expressed. The diversity of functions contributed by the horizontally transferred genes is likely an important factor in the adaptation and evolution of *A*. *queenslandica*. These findings highlight the potential importance of HGT on the success of sponges in diverse ecological niches.

## Introduction

Horizontal gene transfer (HGT) is the acquisition of genes from an exogenous source. HGT among prokaryotes is well-established [1] and examples of HGT from prokaryotes into plants have been reported [2, 3]. HGT in bacteria is important for the evolution of many traits, including metabolic properties and antibiotic resistance [4]. Examples of HGT into other eukaryotes are far fewer, likely due to sequestration of the germline, but may be enhanced by association with organelles, intracellular endosymbionts, parasites, or the presence of active transposable elements [5]. Nevertheless, advances in sequencing technology have made it possible to identify more instances of HGT, particularly into sessile marine invertebrate genomes [6, 7]. Although HGT is rare, the discovery of HGT events in metazoan genomes suggests that acquisition of novel genes from the environment may contribute to biochemical diversification during animal evolution [8]. Widespread HGT from intracellular prokaryotes into eukaryotes have been reported in invertebrates [9–12]. Substantial prokaryotic and eukaryotic gene transfers are also found in the eukaryotic genomes of *Monosiga brevicollis* (choanoflagellate), *Nematostella vectensis* (sea anemone) and *Adineta vaga* (bdelloid rotifer) [13–15]. In bdelloid rotifers, which are asexual metazoans, over 8% of the genes are of bacterial or fungal origin, including enzymes involved in bacterial cell wall peptidoglycan biosynthesis. This suggests that, at least in the rotifer, HGT provides opportunities for gene renewal, sufficient to replace that of sexual reproduction [13]. Other notable examples of HGT in marine organisms include a class of genes for cellulose metabolism in a tunicate [16], genes for aromatic amino acid synthesis and toxin genes in *N*. *vectensis* [17, 18], a mitochondrial DNA repair gene in octocorals [19], and plastid genes into the genome of *Symbiodinium minutum* [20]. Eukaryote to eukaryote transfers have also been documented, including the horizontal transfer of group I mitochondrial introns between sponge and coral species [21], lectin-like antifreeze proteins in fish [22], carotenoid biosynthesis genes from fungi to pea aphids [23], and the transfer of genetic material between chloroplasts of different heterokont species [24].

Poriferans, or sponges, are early diverging metazoans. The mature form is a sessile, benthic, filter feeder. Their simple body plan consists of cells surrounding water canals that filter seawater for food bacteria [25]. Sponges propagate through both asexual and sexual reproduction with germ cells derived from dedifferentiation of pluripotent somatic cells [26, 27]. Sponge tissues play host to many microorganisms and other symbionts, some of which have been shown to produce secondary metabolites that are thought to confer protection against harmful organisms but have also been found to demonstrate a wide range of pharmacological properties [28].

The genome of the demosponge, *A*. *queenslandica*, has recently been sequenced to reveal a rich repertoire of genes very similar to bilaterians [29]. Alignment to known sequences in the UniProt and the NCBI non-redundant databases shows that a percentage of sponge genes exhibit higher similarity to prokaryotic sequences, which is verified through phylogenetic analysis. Although there are proteobacterial symbionts in this sponge, further analysis of the genomic locations and nucleotide characteristics of the prokaryote-like genes suggests that some might have been gained through horizontal gene transfer. In this study, we aimed to identify which of these genes were most likely transferred from prokaryotic donors. We found that the *A*. *queenslandica* genome exhibits evidence of multiple putative horizontal gene transfer events. Candidate horizontally transferred genes encode a variety of enzymes, which may provide an evolutionary advantage by conferring adaptability of sponges to diverse environments.

## Results

### Identifying putative HGT events

Several lines of evidence are typically used to detect potential HGT events, including patchy phyletic distribution of a gene [30, 31], atypical intron features [32, 33], or atypical nucleotide composition and codon usage patterns [34, 35]. However, it is generally recognized that all HGT detection methods have limitations and it is thus recommended that several methods be compared to infer that a HGT event has occurred [35–37]. To identify putative HGT events in the *A*. *queenslandica* genome, we used four independent methods. First, we used Alien Index (AI) analysis to identify genes with greater similarity to prokaryotic sequences [13]. Second, we performed Blastp of *A*. *queenslandica* predicted peptides against the NCBI non-redundant database to identify proteins that align best to prokaryotic sequences. Third, we used EvolMAP, an algorithm that infers the gene composition of multiple ancestral genomes through the use of a species tree-based gene clustering method [38]. And fourth, to complement the protein based searches above, which are focused on finding HGTs that have evolved into functional eukaryotic genes, we utilized a nucleotide sequence-based method that recognizes younger HGTs that retain a prokaryotic nucleotide signature [39]. Because sponges are known to harbor many prokaryotic symbionts, we implemented additional filters based on GC content, location within the sponge genome assembly, and average expression to minimize the detection of genes that may represent sequences from symbionts.

**Fig 1:**
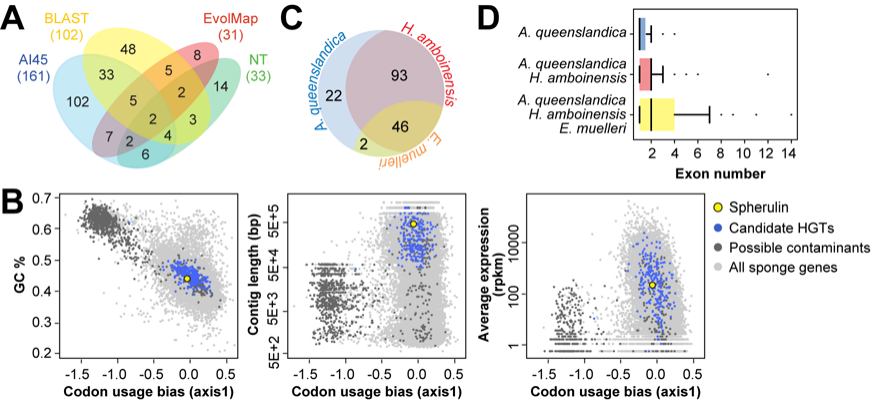
Putative horizontally transferred genes in the genome of *A*. *queenslandica*. (A) The number of candidate HGTs detected by Alien Index (AI45), Blast, EvolMAP, and the nucleotide pipeline (NT). (B) Comparison of compositional traits of putatively transferred genes and host genes. Scatterplots show primary codon usage plotted against the GC content of protein coding genes (GC%), the size of the genome contig on which the gene is located, and the average expression in sponge larvae and adult tissues (reads per kilobase per million, rpkm). Candidate HGTs, blue; potential contaminants, dark grey; spherulin, yellow; other sponge genes, light grey. (C) The number of putative horizontally transferred genes in *A*. *queenslandica* with homology to sequences in the demosponges, *H*. *amboinensis* and *E*. *muelleri*. (D) Boxplots showing the exon number distribution for horizontally transferred genes with homologs in the indicated sponge lineages.

A total of 227 putative HGT events (HGT set), some of which were identified by multiple methods, were detected in the *A*. *queenslandi*ca genome (Fig 1A, S1 Table). Thirty one candidate HGTs were identified by EvolMAP, 102 by Blast, 161 by AI45, and 33 by the nucleotide-based pipeline. Blast and AI45 detected many genes in common, which is not surprising as both of these methods rely on alignment to the NCBI non-redundant protein database to detect potential HGTs. However, additional manual inspection of Blast alignments for eukaryotic matches reduced the total number of overlapping genes to 37. On the other hand, EvolMAP detected only 14 genes in common with Blast and 16 with AI45, most likely because it uses a different protocol and criteria for detecting HGT events. The nucleotide pipeline discovered 33 putative HGTs, 19 of which were also identified by at least one of the protein-based methods.

### Verifying putative HGTs

Horizontally transferred genes that have been integrated into a genome for some time assimilate the compositional traits of the host to promote gene expression and processing [40]. Diagnostic features include the presence of introns, similarity in overall GC content, and similarity of codon usage. The selection of a subset of optimal codons allows for translational efficiency, particularly for genes that are more highly expressed. Thus, the codon usage pattern of a genome is a characteristic feature of an organism or taxonomic group and can be used to study selection pressure and mutational bias [41–43]. Unusual codon usage or GC content has been used as a way to distinguish gene transfer in prokaryotes [34, 35], as well as in eukaryotes [37, 44, 45].

To determine whether the four HGT detection methods used in this study are able to differentiate between potential HGTs, which have assimilated into the host genome, versus symbiont genes, we performed correspondence analysis of codon usage using CodonW (J. Peden, Nottingham, UK). We then compared the compositional characteristics of genes within the clusters that are distinguishable by codon usage bias. This analysis revealed that *A*. *queenslandica* genes could be clustered into two major groups with variable codon usage. Plotting codon usage bias against the GC content (GC%) revealed that genes eliminated by the HGT filters fall into the outlier cluster with higher GC content and divergent codon usage values (Fig 1B). Similarly, we observed that genes in the outlier cluster are mostly located on contigs smaller than 50kb. Variable codon usage characteristics, coupled with divergent GC content and location on smaller assembled contigs, suggest that genes in the outlier cluster are most likely derived from sponge-associated prokaryotes or, alternatively, from very recently transferred genes. Candidate HGTs that are found on larger assembled contigs and that exhibit similar codon usage and GC content as the majority of sponge genes are possibly prokaryotic genes that have become assimilated into the sponge genome. It is important to note, however, that the shorter contigs of the sponge genome assembly may also represent difficult-to-assemble sequences, such as repeats and GC-rich regions.

Active transcription of a horizontally acquired gene provides the first line of evidence that a gene is functional in its new host [6]. However, it should be kept in mind that methods to detect expression, such as transcriptome analysis, might also detect expressed transcripts from symbiotic or contaminating bacteria present in the original samples, and that genes that are conditionally or specifically expressed will not be detected in single-stage or single-tissue samples. Analysis of gene expression revealed that putative horizontally transferred genes in *A*. *queenslandica* exhibit a wide range of abundance in both larvae and adult sponge tissues. As further validation of our methods, we observed that the spherulin gene, which has been reported to be horizontally transferred in the sponge [44], clusters with the other candidate HGTs in *A*. *queenslandica*.

Further support that the HGT candidates are incorporated into the functional genome of *A*. *queenslandica* comes from examination of sequences from other sponge species. Many of the putative horizontally transferred genes in *A*. *queenslandica* have detectable homologs in the transcriptomes of other sponges, including another marine haplosclerid demosponge, *Haliclona amboinensis* [46], and the freshwater demosponge, *Ephydatia muelleri* [47] (Fig 1C). This suggests that these prokaryote-like genes may have been ancient transfers into the demosponge lineage. Although it is possible that some candidate HGTs are derived from common sponge symbionts that were sequenced along with the sponge transcriptomes, the finding that genes with homologs in both *H*. *amboinensis* and *E*. *muelleri* possess more exons compared to genes that are found only in *A*. *queenslandica* (Fig 1D) lends further support to the hypothesis that these genes were transferred from prokaryotes and have ameliorated to the nucleotide composition and gene structure of their host over time.

It is often difficult to identify the donor species for putative HGTs using sequence similarity because the transferred genes may not yet be represented in available databases or because they have resided in the sponge long enough to diverge and integrate host genome characteristics. Nevertheless, some putative sponge HGTs show detectable similarity (e-value < 1x10^-5^) but with low percent identity to genes in various marine bacteria, including *Vibrio campbellii*, which is present as both a free-swimming bacterium in tropical marine waters or as a commensal in the gut microflora of marine animals, *Desulfovibrio hydrothermalis*, a sulfate reducing bacteria discovered in hydrothermal vents, and *Arcobacter nitrofigilis*, a symbiotic bacteria in the marine environment (S2 and S3 Tables). The sequencing of additional prokaryotic genomes, particularly of symbionts of marine organisms, may eventually allow the identification of the donors of candidate HGTs in the sponge genome.

### Classification of candidate HGTs

**Fig 2:**
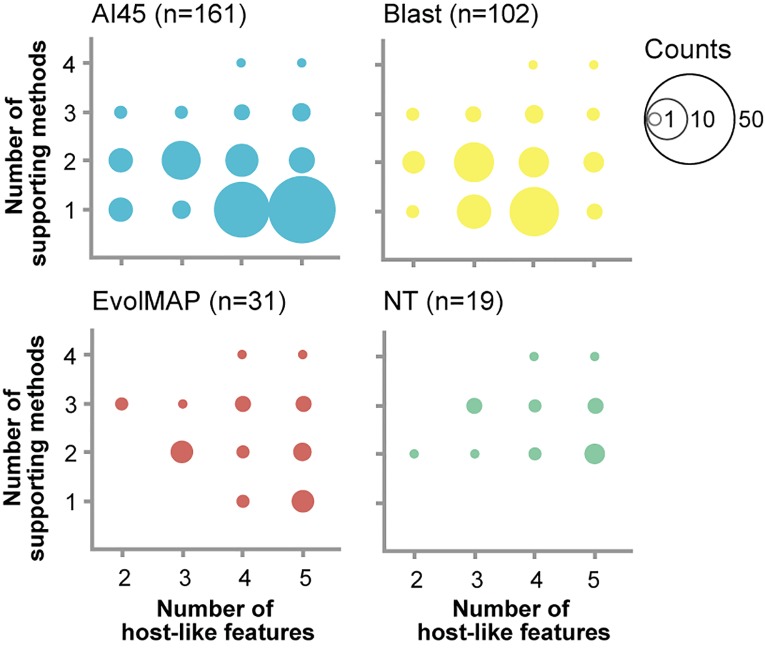
Classification of putative horizontally transferred genes based on number of supporting methods and host-like features. AI45, Blast, EvolMAP, and the nucleotide pipeline (NT) detect candidate HGTs with distinct characteristics, suggesting that these methods have different sensitivities for identification of young versus old HGTs or for discriminating against contaminating prokaryotic sequences. Only candidate HGTs that passed the additional criteria for location on larger contigs and expression in sponge tissues were included in this analysis (numbers in parentheses). Sizes of the circles represent the number of genes under each classification.

Genes identified as putatively acquired from prokaryotic donors can be classified according to two metrics based on different criteria. The “number of supporting methods” provides an estimate of how likely a gene is to be a *bona fide* HGT. This metric is based on detection of the gene by the four independent methods used in the study (Fig 2, S1 Table). The “number of host-like features” provides an estimate of how long a gene has been residing in the host genome. This metric is based on the degree of assimilation of host genome characteristics (GC%, codon usage bias, presence of multiple exons), as well as the presence of identifiable homologs in other sponge lineages (*H*. *amboinensis* and *E*. *muelleri*). However, because only transcriptome data is available for these other demosponges, the absence of homologs for some genes may also reflect lack of expression in the biological material that was subjected to transcriptome sequencing. Nevertheless, it is interesting to note that the various methods detect different sets of putative HGTs with varying numbers of host-like features. EvolMAP and the nucleotide-based method identified the fewest putative HGTs, many of which were also supported by other methods and exhibited more host-like features, indicative of higher confidence horizontally transferred genes. On the other hand, AI45 and Blast detected many putative HGTs that were not found by the other methods. AI45 genes tend to exhibit more host-like features compared to those discovered by protein Blast. This suggests that the Blast method may identify transferred genes that have not yet had time to assimilate host genome characteristics or, alternatively, that it identifies more contaminating prokaryotic sequences.

Interestingly, the nucleotide sequence-based pipeline, which is more likely to detect relatively “young” HGTs that have retained a signature of prokaryotic origin at the nucleotide level, can also detect ancient HGTs that have evolved into functional eukaryotic genes [39]. Following manual curation, the nucleotide pipeline identified 33 HGT candidates. Nineteen of these overlap with genes that were also identified using the protein sequence-based methods, thus providing further support for these as having been acquired from prokaryotic donors (S1 and S3 Tables). It is important to note that the nucleotide signal degrades over time due to mutation and selection. Hence, the nucleotide pipeline is geared towards detecting more recent HGTs while the protein pipeline is more likely to detect ancient HGTs that may have lost the prokaryotic signature at the nucleotide level. It is surprising that the nucleotide pipeline did not detect more HGTs, as they are routinely found by this approach in terrestrial arthropod genomes [9, 39]. The finding suggests that ongoing HGT events may be rare in *A*. *queenslandica*. Alternatively, a current paucity of sequenced genomes of microbes associated with aquatic invertebrates (e.g. symbionts) could reduce effectiveness of the pipeline to detect recent HGTs from such sources.

### HGT functions and contribution to sponge adaptability

**Fig 3:**
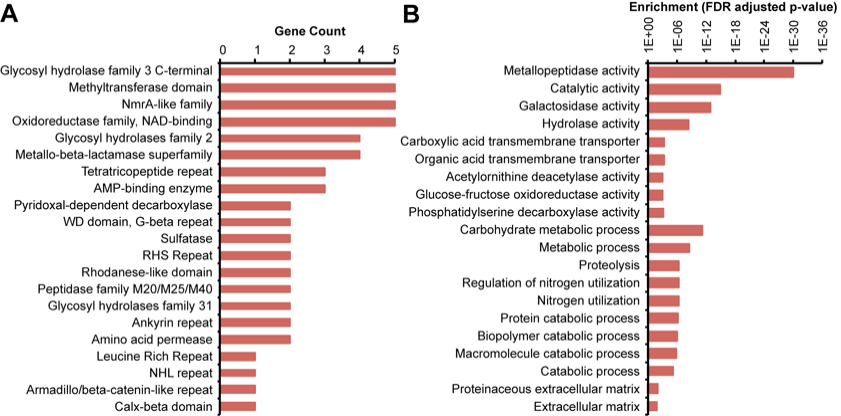
Functions represented in putative horizontally transferred genes in the *A*. *queenslandica* genome. (A) Number of putative horizontally transferred genes containing the indicated PFAM protein domains. (B) Gene ontology analysis for putative horizontally transferred genes in *A*. *queenslandica*. Enrichment p-values for selected functions of putative horizontally transferred genes are shown.

Horizontally acquired genes in bacteria tend to be catalytic and typically encode enzymes involved in metabolism [48]. Of the putative horizontally transferred genes in *A*. *queenslandica*, only 71% (162 out of 227) have matches in UniProt, gene ontology annotation, or a recognizable PFAM domain. The remaining 29% are of unknown function. Of the genes with recognizable PFAM domains, 51% (53 out of 104) possess only a single domain, which is characteristic of prokaryotic genes. Catalytic domains are present in 76 genes, indicating that horizontal transfer can confer enzymatic activities. The most common PFAM domains represented within the HGT set include glycosyl hydrolase (carbohydrate metabolism), methyltransferase (protein modification), NmrA-like family (nitrogen metabolite repression), oxidoreductase, metallo-beta-lactamase (breakdown of antibiotics), and sulfatase (hydrolysis of sulfate esters in a wide range of biomolecules) (Fig 3A). Complementary analysis using Gene Ontology (GO) reveals that the most enriched functions in the set of putative horizontally transferred genes involve catalysis and metabolism of various biomolecules, including carbohydrates and proteins (Fig 3B). Metallopeptidase activity is the most enriched gene ontology term. The enrichment of catalytic functions in candidate horizontally transferred genes is consistent with reports that operational genes, such as biosynthetic enzymes, are more frequently transferred than informational genes, such as transcription and translation factors, which are part of large interaction networks [48]. This also indicates that gene transfer may be a source of novel enzymatic functions for alternative metabolic activities in the host, such as the production of bioactive compounds [28].

### Examples of prokaryotic gene transfers maintained in the sponge genome

The validity of our method in detecting HGTs is further supported by the detection of ancient transfers from prokaryotes that have been maintained in the sponge lineage. However, our method also detects more recent transfers maintained in a closely related sponge species also belonging to the suborder Haplosclerida.

#### Spherulin

**Fig 4:**
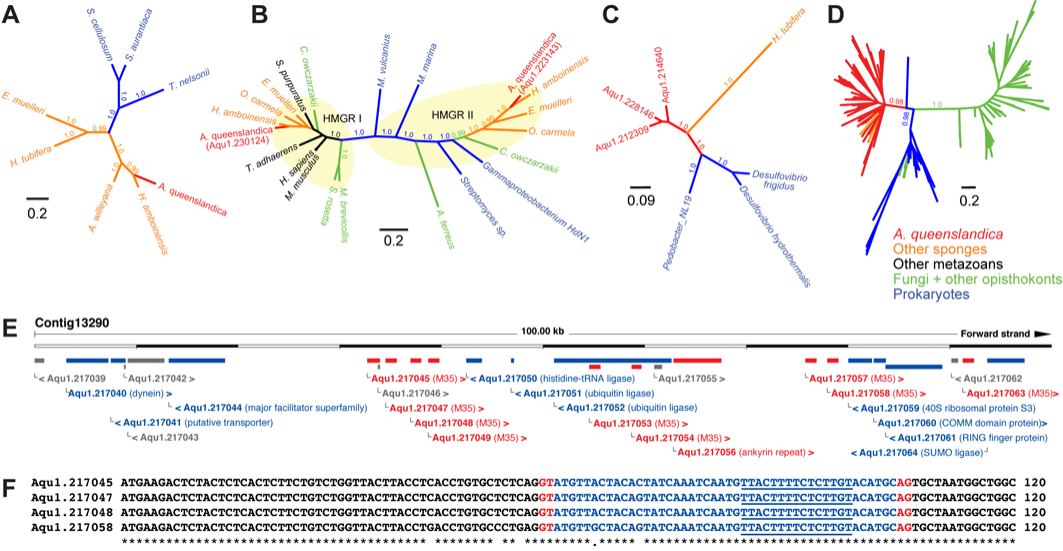
Examples of candidate horizontally transferred genes in the *A*. *queenslandica* genome. Unrooted phylogenetic trees depicting the relationship of (A) spherulin, (B) 3-hydroxy-3-methylglutaryl-CoA reductase (HMGR), (C) phosphatidylserine decarboxylase (PSD), and (D) M35 metallopeptidase sequences from *A*. *queenslandica* (red) to sequences in other sponges (orange), prokaryotes (blue), fungi or opisthokonts (green), and other metazoans (black). Trees were generated using MrBayes with posterior probabilities depicted on selected branches. Support values on some branches and tip labels in (D) were omitted for clarity. Sequences for spherulin, HMGR, and PSD genes were obtained by Blastp searches in NCBI or Compagen [47]. Prokaryotic and fungal metallopeptidase sequences were obtained from MEROPS [49]. (E) Example of a cluster of prokaryote-like genes (red) flanked by metazoan-like genes (blue) on an *A*. *queenslandica* contig. Genes are labeled with the name of their best hit in the UniProt database; metallopeptidases are labeled M35. Genes of unknown origin are shown in grey. The figure was adapted from Ensembl. (F) Alignment of partial sequences for selected metallopeptidase genes showing the presence of introns (blue) with canonical splice sites (red) and polypyrimidine tract (underlined).

Spherulin was detected as a prokaryotic gene transfer into the coralline demosponge, *Astrosclera willeyana* [44]. This gene is highly expressed in sponge spherulite-forming cells, suggesting a role in biocalcification. The spherulin gene is also present in *A*. *queenslandica* and other demosponges but is absent outside of the demosponge group, suggesting that the horizontal transfer event occurred specifically in this lineage (Fig 4A).

#### Isoprenoid synthesis

Isoprenoids are a large family of compounds synthesized by all free-living organisms. They serve numerous functions, including major structural components of cell membranes, hormones, and plant defense compounds [50, 51]. Two independent non-homologous metabolic pathways for isoprenoid biosynthesis are known: the methylerythritol phosphate pathway in bacteria and the mevalonate pathway in eukaryotes and archaea [52]. The rate-controlling step of the mevalonate pathway is the 3-hydroxy-3-methylglutaryl-CoA reductase (HMGR) enzyme, which catalyzes the conversion of hydroxymethylglutaryl-CoA into mevalonate. Two classes of HMGR genes have been identified. Class I HMGR is present in eukaryotes, archaea, some proteobacteria and actinobacteria, while the class II is characteristic of most bacteria and a small number of archaea [53]. While these two classes of enzymes share similar active sites, they possess different domain structures and are differentially regulated by cofactors and statins [54].

Interestingly, all demosponges interrogated in this study possess two HMGR genes whereas other metazoans only have one. Phylogenetic analysis of HMGR sequences revealed that one of the two genes from each sponge clusters with the prokaryotic HMGR sequences (Fig 4B) while the other clusters with HMGR genes from other metazoans. Both HMGR classes are present in the filasterean *Capsaspora owczarzaki* and class II HMGR genes can be found in some fungi. These findings suggest that the class II HMGR may have been transferred from a prokaryotic donor into the opisthokont ancestor and has been maintained in sponges but lost in other metazoans.

Actinobacteria possessing HMGR have been reported to produce novel isoprenoids via the mevalonate pathway [55]. Thus, the maintenance of two enzymes involved in isoprenoid biosynthesis in sponges suggests the potential for the production of diverse small molecule precursors of compounds that are presumably used by the sponge for defense against competitors and predators, such as sphingosine derivatives in *Haliclona vansoesti* [56] and terpene isocyanides in *Amphimedon terpenensis* and *Axinyssa* [57].

#### Phosphatidylserine decarboxylases

Phosphatidylethanolamine (PE) is an abundant membrane phospholipid. In bacteria, yeast, plant, and animal cells, this phospholipid is formed by decarboxylation of phosphatidylserine through the action of phosphatidylserine decarboxylases (PSDs) [58]. Yeast, plant, and mammalian cells further possess an alternative pathway for the synthesis of PE. In the *A*. *queenslandica* genome, three PSD genes were identified as putative HGTs (Fig 4C) through the AI45 method, as well as by the nucleotide sequence-based pipeline. A homologous gene was identified in the transcriptome of the marine haplosclerid demosponge, *Haliclona tubifera*. The maintenance of PSDs in all domains of life emphasizes their importance in lipid metabolism and membrane biogenesis.

#### Extracellular peptidases

The *A*. *queenslandica* genome has 76 metallopeptidase domain-containing genes belonging to the M35 family. This family of peptidases has been detected in many bacterial and fungal genomes, but not in metazoan genomes, except for the sponge [49]. 56 of these are potential HGTs detected by either the Blast method or AI45. Phylogenetic analysis of the sponge metallopeptidase domains reveal that the sponge genes are more closely related to bacterial peptidase genes (Fig 4D). Selected metallopeptidase sequences from *H*. *amboinensis* cluster with the *A*. *queenslandica* sequences further suggesting that these were ancient transfers into the sponge lineage. In the *A*. *queenslandica* genome, some of the peptidase genes appear in clusters on assembled genomic contigs, suggesting tandem duplication (Fig 4E). Most of the genes exhibit assimilation of sponge genome characteristics, including the presence of canonical spliceosomal introns (Fig 4F). Bacterial M35 metallopeptidases are known virulence factors and extracellular peptidases [59–61], while different families of animal metallopeptidases are important players in extracellular matrix remodeling, particularly during embryonic development [62]. Thus, while the actual roles of these prokaryotic metallopeptidase-like genes in the sponge remain to be explored, it is hypothesized that they may serve a protective function, as a defense against non-symbiotic bacteria or other organisms. Alternatively, they may be involved in the plasticity and maintenance of the proteinaceous extracellular matrix in which sponge cells are embedded.

## Discussion

Sequencing of the *A*. *queenslandica* genome revealed multiple bacterial sequences that may be derived from proteobacterial symbionts [29]. Using diverse criteria, including sequence features and gene expression patterns, we now conclude that many ancient prokaryote genes reside in the *A*. *queenslandica* genome and are likely the result of multiple horizontal transfers. This is not unprecedented, as there have been previous reports of massive transfers of prokaryotic sequences into eukaryotes, such as the bdelloid rotifer and filamentous eukaryotes [13, 63]. Once transferred, prokaryotic genes acquire introns and adapt the compositional traits of their host genome. These genes are expressed and become integrated into eukaryotic cellular processes or confer a novel function for the host.

Sponges do not have specialized reproductive organs. Germ cells are derived from pluripotent somatic cells [27], which may come in contact with prokaryotes. Furthermore, sponges release sperm into the water and currents carry the sperm to the archaeocytes of another sponge for fertilization. Thus, sexual reproduction in the sponge provides multiple potential entry points to the germ line for foreign DNA. However, a specific donor for any horizontally transferred gene is difficult to ascertain due to the substantial divergence of sequences or the lack of representatives in current databases. Nevertheless, some of the genes we have identified show similarity to sequences from various marine bacteria.

Genes that have undergone horizontal transfer may extend an organism’s phenotype contributing to critical features of the organism’s morphology, lifestyle, and behavior [6]. For example, the spherulin gene of *A*. *willeyana* was transferred from bacteria and became a key component of the sponge biomineralization strategy [44]. Gene ontology analysis of HGT candidates shows enrichment for catalytic activity, with the metallopeptidases and galactosidases as the largest categories. Thus, HGT may be a source of diverse catalytic functions that have been harnessed by the sponge to produce a repertoire of complex biochemical compounds. One could further speculate that the horizontal transfer of multiple carbohydrate and protein catalytic enzymes into the sponge lineage may contribute to establishing their complex cellular architecture and to implementing the highly dynamic cellular relationships within these organisms.

Although a recent study suggests that the cumulative effect of horizontal gene transfers in eukaryotic genomes is small and that most prokaryotic genes were acquired in two events through mitochondrial and plastid transfer [64], in the sponge genome we see a larger number of genes transferred with some functional relationships among them related to modification of cellular membranes and the extracellular matrix. Thus, our findings highlight the potential importance of HGT in sponge adaptation and evolution and in their success in colonizing diverse ecological niches.

Reannotation of the genome of *A*. *queenslandica* using data from deep developmental transcriptomes revealed even more previously undiscovered genes [65]. The new annotation retained 86% of originally predicted sponge genes, including 214 of the 227 candidate HGTs identified in this study (S1 Fig). The inclusion of developmental transcriptome data improved gene model prediction and likely eliminated genes derived from contaminants, as evidenced by the decreased representation of gene models common to both annotation versions in the codon usage outlier cluster (S1 Fig). Analysis of compositional characteristics revealed that most of the gene models unique to the new annotation share similar nucleotide features as the majority of sponge genes (S2 Fig). However, because many of these new gene models share no similarity to sequences in the NCBI NR database, their affiliation with prokaryotic or eukaryotic groups is yet to be verified. While it remains challenging to identify horizontally acquired genes in basal metazoans, the extent and significance of HGT in sponges and other eukaryote genomes will become more apparent as further careful evaluation of prokaryotic sequences discovered in eukaryotic sequencing projects is conducted.

## Materials and Methods

### Identification of HGTs by Alien Index

*A*. *queenslandica* genome sequences were downloaded from Ensembl Metazoa (Aqu1.20 version) [29]. Predicted peptides were aligned by Blastp against the NCBI non-redundant (NR) database. The Alien Index (AI) for each protein was computed as the log-transformed difference between the best Blastp e-value to a metazoan hit and the best Blastp e-value to a non-metazoan hit in NR [13]. Comparison of metazoan versus prokaryote best Blastp hit e-values reveals that genes with an AI≥45 exhibit closer similarity to prokaryotic sequences (S3 Fig). Thus, only *A*. *queenslandica* genes with an AI≥45 (AI45) and a best Blast hit against a prokaryotic sequence were selected for further analysis of potential HGTs from prokaryote donors.

### Identification of HGTs by Blast alignment

Predicted *A*. *queenslandica* peptides were aligned against the NCBI NR protein database using Blastp with a threshold e-value of 1x10^-5^ [66]. The taxon affiliation of sequence matches was determined using the NCBI Taxonomy Database. Genes were assigned to the consensus phylum of their top 10 sequence matches. The Blastp alignments for genes assigned to phylum Bacteria were manually inspected to confirm that they have no significant similarity to eukaryotic and metazoan sequences. Only genes that matched exclusively to prokaryotic sequences were selected for further analysis.

### Identification of HGTs by EvolMAP

EvolMAP analysis was used to find gene families that show common ancestry in prokaryotes and *A*. *queenslandica*. This method infers the composition of multiple ancestral genomes through the use of a species tree-based gene clustering method [38]. EvolMAP was conducted using genomes from 17 eukaryotes representing major phyla of unicellular and multicellular organisms, as well as 45 prokaryotes (7 Archaea and 38 Bacteria) representing major phyla within the prokaryotic domain (S4 Fig, S4 Table). Gene families that are conserved between *A*. *queenslandica* and prokaryotes, but not in other eukaryotes or metazoans, were selected as putative HGTs for further analysis.

### Nucleotide sequence-based HGT pipeline

To facilitate detection of prokaryotic HGTs embedded in eukaryotic contigs, the *A*. *queenslandica* genome was broken down into 1kb intervals and searched using Blastn against a prokaryotic database containing about 1000 species and masked for low complexity regions using the NCBI Dustmasker function [9, 39]. Given the fragmented nature of the sponge genome assembly and the general nucleotide characteristics of its smaller contigs, we focused on contigs >50kb to increase the chances of detecting *bona fide* HGTs. To provide a breadth of taxa for screening of candidate HGTs, the set of target prokaryotic genomes (1095 species from 565 genera [39]) were selected to include representatives from 27 of 30 bacteria phyla (http://www.bacterio.net/-classifphyla.html) and 5 of 5 archaea phyla. Any 1kb fragment found to contain a prokayotic hit (bit score) greater than the eukaryotic score was harvested. Fragments with positive hits were then searched against a eukaryotic genome database containing representatives from the following genera: *Anopheles*, *Apis*, *Drosophila*, *Xenopus*, *Tribolium*, *Nasonia*, *Daphnia*, *Strongylocentrotus*, *Mus*, *Homo*, *Aplysia*, *Caenorhabditis*, *Hydra*, *Monosiga and Acanthamoeba* (S4 Table). The eukaryotic database was used to detect and screen out highly conserved genes that are shared between prokaryotes and eukaryotes. A significance e-value threshold of 1x10^-5^ was used for both the eukaryotic and prokaryotic hits. To focus on stronger candidates, only regions of prokaryotic similarity greater than 100bp and with bit score difference between prokaryote and eukaryote >25 were manually curated using Blastn and Blastx against the NCBI NR database to determine its validity.

### Post-filtering of putative HGTs

To remove potentially contaminating sequences from prokaryotic symbionts of the sponge, candidate horizontally transferred genes were further screened to eliminate (1) genes with a GC content at codon positions 1 and 3 (GC13%) greater than two standard deviations from the majority of sponge genes, (2) genes located on *A*. *queenslandica* genome contigs less than 50kb, and (3) genes with an average expression lower than 10 reads per kilobase per million (rpkm) in larval and adult developmental stages based on available transcriptome data [67]. Moreover, candidate genes were examined manually to determine whether they are found on sponge genome contigs with flanking metazoan genes on either side, indicative of valid HGTs.

### Correspondence analysis of codon usage

To measure the degree to which sponge genes have adapted towards the use of optimal codons, correspondence analysis of codon usage was performed using CodonW (J. Peden, Nottingham, UK). Codon usage bias represents the primary orthogonal axis that explains the greatest variation in codon usage within the data and reveals the most robust patterns or differences among the genes.

### Classification of candidate HGTs

The ‘number of supporting methods’ for a putative HGT is the sum of the number of independent methods that flag it as potentially of prokaryotic origin (maximum = 4). The ‘number of host-like features’ for a putative HGT is calculated as the sum of the number of features that it shares with the host (GC%, codon usage, exon number), as well as the presence of homologs in transcriptomes of other sponge species (S4 Table), including a closely related marine haplosclerid demosponge (*H*. *amboinensis*) and a more distantly related freshwater demosponge (*E*. *muelleri*) (maximum = 5).

### Gene ontology analysis

*A*. *queenslandica* predicted peptides were aligned to proteins in the UniProt database using Blastp with an e-value cutoff ≤ 1x10^-4^. Genes were assigned the names and gene ontology annotations of their best match. Gene ontology term enrichment was estimated using topGO [68]. Enrichment of genes in specific functional groups was determined using Fisher’s exact test. The statistical significance threshold was corrected for multiple hypothesis testing using the Benjamini-Hochberg method.

### Phylogenetic analysis

Homologous protein sequences were identified by Blastp against the NCBI NR database and transcriptome resources for selected sponge species (S4 Table). Sequences were aligned using ClustalW2 [69] then trimmed using Gblocks [70]. Phylogenetic analyses were conducted using MrBayes 3.2.2 [71] with two independent MCMC runs and four chains per run. Each analysis set for 1 million generations sampled every 100 trees or until the standard deviation of split frequencies was <0.01. The first 25% of trees were discarded as burn-in.

## Supporting Information

S1 Fig Majority of Aqu1 candidate HGTs are represented in the Aqu2 annotation of the *A*. *queenslandica* genome. Scatterplots show the primary codon usage axis plotted against the GC content (GC%) of protein coding genes or the size of the genome contig on which the gene is located for genes in both Aqu1 and Aqu2 annotations (A-B, red circles) or genes found only in Aqu1 (C-D, blue circles). Interestingly, 14% of Aqu1 gene models not represented in Aqu2 fall within the codon usage bias outlier cluster (dashed circle) compared to only 2% of common gene models. Other Aqu1 genes are indicated by grey circles. (E) Scatterplot of primary codon usage axis plotted against the GC content for HGT candidates found in both Aqu1 and Aqu2 (red) or only in Aqu1 (blue). (F) The number of candidate HGTs also represented in Aqu2 that are detected by Alien Index (AI45), Blast, EvolMAP, and the nucleotide pipeline (NT). (G) The number of candidate HGTs represented in Aqu2 with homology to other demosponges. (H) Boxplots of the revised exon number distribution for candidate HGTs represented in Aqu2.
(TIF)

S2 Fig Analysis of new gene models in the Aqu2 annotation. Scatterplot of primary codon usage axis plotted against the GC content (A-C) or contig length (B-D) for all Aqu2 gene models (A-B) or for gene models found only in Aqu2 (C-D). HGT candidates in Aqu2, based on AI≥45, are shown in red. Other Aqu2 genes are shown in grey. Only 4.5% of all Aqu2 gene models fall within the codon usage bias outlier cluster (dashed circle) compared to 13% of gene models unique to Aqu2.
(TIF)

S3 Fig Blastp e-value distribution for genes with Alien Index ≥ 45. Comparison of the e-values for the best metazoan hit and best bacteria hit for sponge genes reveals that those with AI≥45 are affiliated more closely with the bacteria axis. To further enrich for potential transfers from prokaryote donors, only genes with AI≥45 and a best Blast hit to a prokaryotic sequence were selected for further analysis (blue, all sponge genes; yellow, AI≥45; red, AI≥45 and a best Blast hit to a prokaryotic sequence).
(TIF)

S4 Fig Species tree used for EvolMAP analysis. Branch lengths represent average ortholog divergence as computed by EvolMAP. The representative eukaryotic and prokaryotic species included in the analysis are shown.
(TIF)

S1 Table List of candidate horizontally transferred genes in the *A*. *queenslandica* genome and their sequence features. The table lists the 227 candidate HGTs identified by the 4 independent methods and that passed all post-filtering criteria. The table also lists 42 genes that passed HGT criteria but are expressed at an average of less than 10 rpkm.
(XLSX)

S2 Table Candidate HGTs detected by EvolMAP and their best prokaryote sequence match based on Blastp against the NR database. (XLSX)

S3 Table Candidate HGTs detected by the nucleotide pipeline and their best Blastn match. (XLSX)

S4 Table Genomes and transcriptomes used in the study. Accession numbers and sources for the genomes and transcriptomes used for EvolMAP analysis, the nucleotide pipeline, as well as for specific gene homology searches are listed.
(XLSX)
