# Supplementary figures and images for: Detection of Prokaryotic Genes in the Amphimedon queenslandica Genome
Source: PLoS One. 2016 Mar 9;11(3):e0151092. doi: 10.1371/journal.pone.0151092 (PMC4784904; doi:10.1371/journal.pone.0151092)

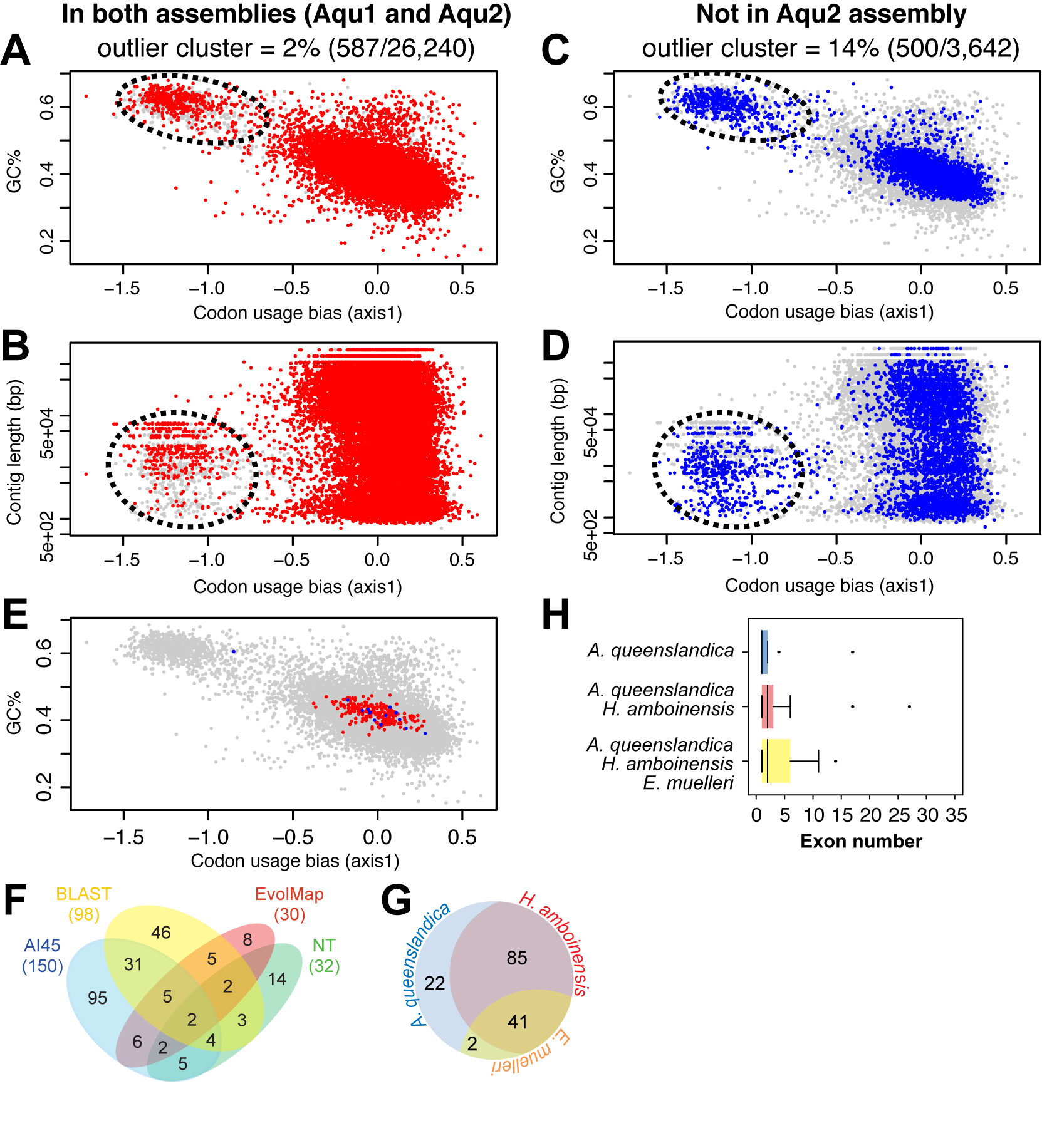

Supplement: S1 Fig — Scatterplots show the primary codon usage axis plotted against the GC content (GC%) of protein coding genes or the size of the genome contig on which the gene is located for genes in both Aqu1 and Aqu2 annotations (A-B, red circles) or genes found only in Aqu1 (C-D, blue circles). Interestingly, 14% of Aqu1 gene models not represented in Aqu2 fall within the codon usage bias outlier cluster (dashed circle) compared to only 2% of common gene models. Other Aqu1 genes are indicated by grey circles. (E) Scatterplot of primary codon usage axis plotted against the GC content for HGT candidates found in both Aqu1 and Aqu2 (red) or only in Aqu1 (blue). (F) The number of candidate HGTs also represented in Aqu2 that are detected by Alien Index (AI45), Blast, EvolMAP, and the nucleotide pipeline (NT). (G) The number of candidate HGTs represented in Aqu2 with homology to other demosponges. (H) Boxplots of the revised exon number distribution for candidate HGTs represented in Aqu2. (TIF) [file pone.0151092.s001.tif]

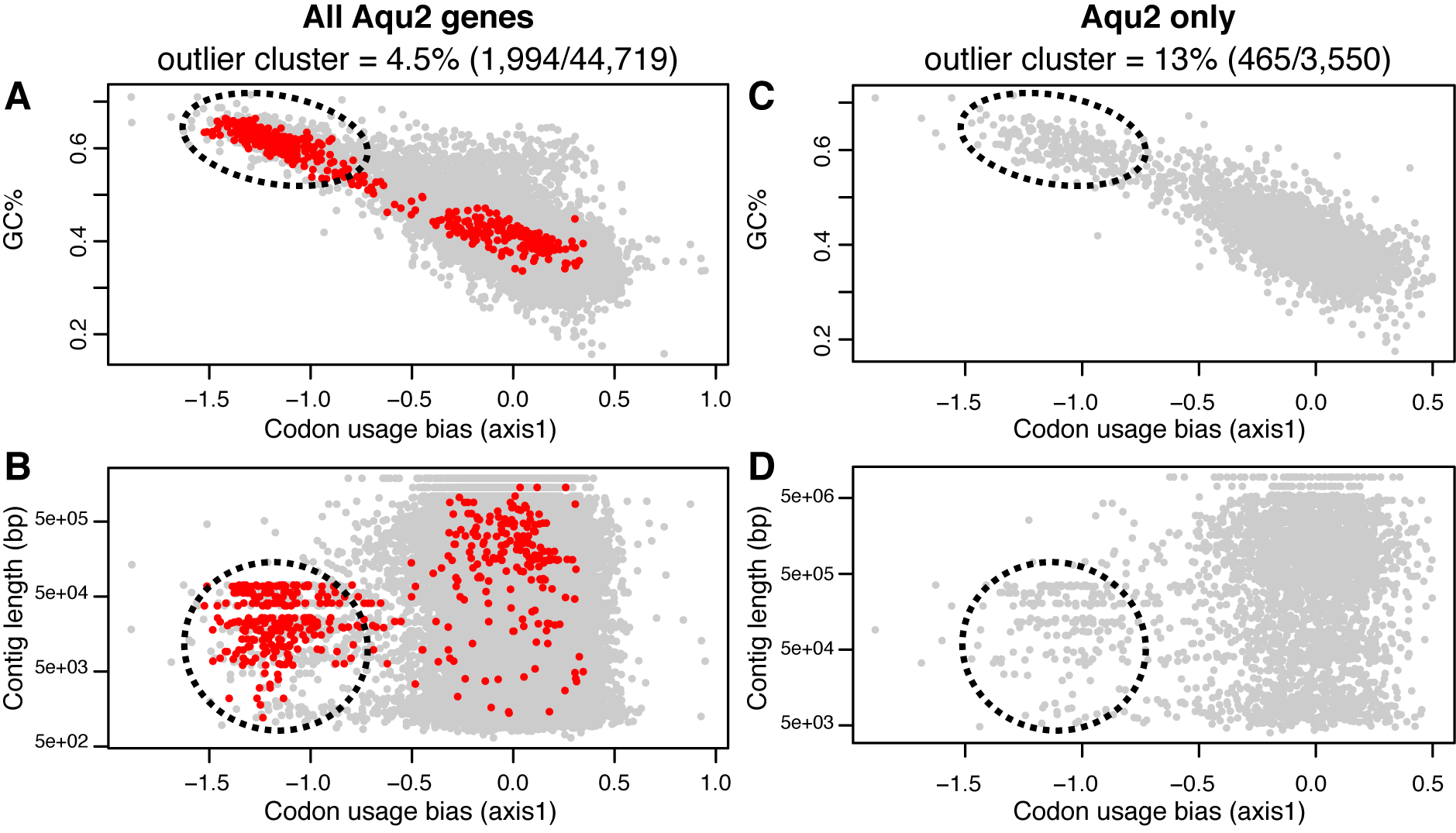

Supplement: S2 Fig — Scatterplot of primary codon usage axis plotted against the GC content (A-C) or contig length (B-D) for all Aqu2 gene models (A-B) or for gene models found only in Aqu2 (C-D). HGT candidates in Aqu2, based on AI≥45, are shown in red. Other Aqu2 genes are shown in grey. Only 4.5% of all Aqu2 gene models fall within the codon usage bias outlier cluster (dashed circle) compared to 13% of gene models unique to Aqu2. (TIF) [file pone.0151092.s002.tif]

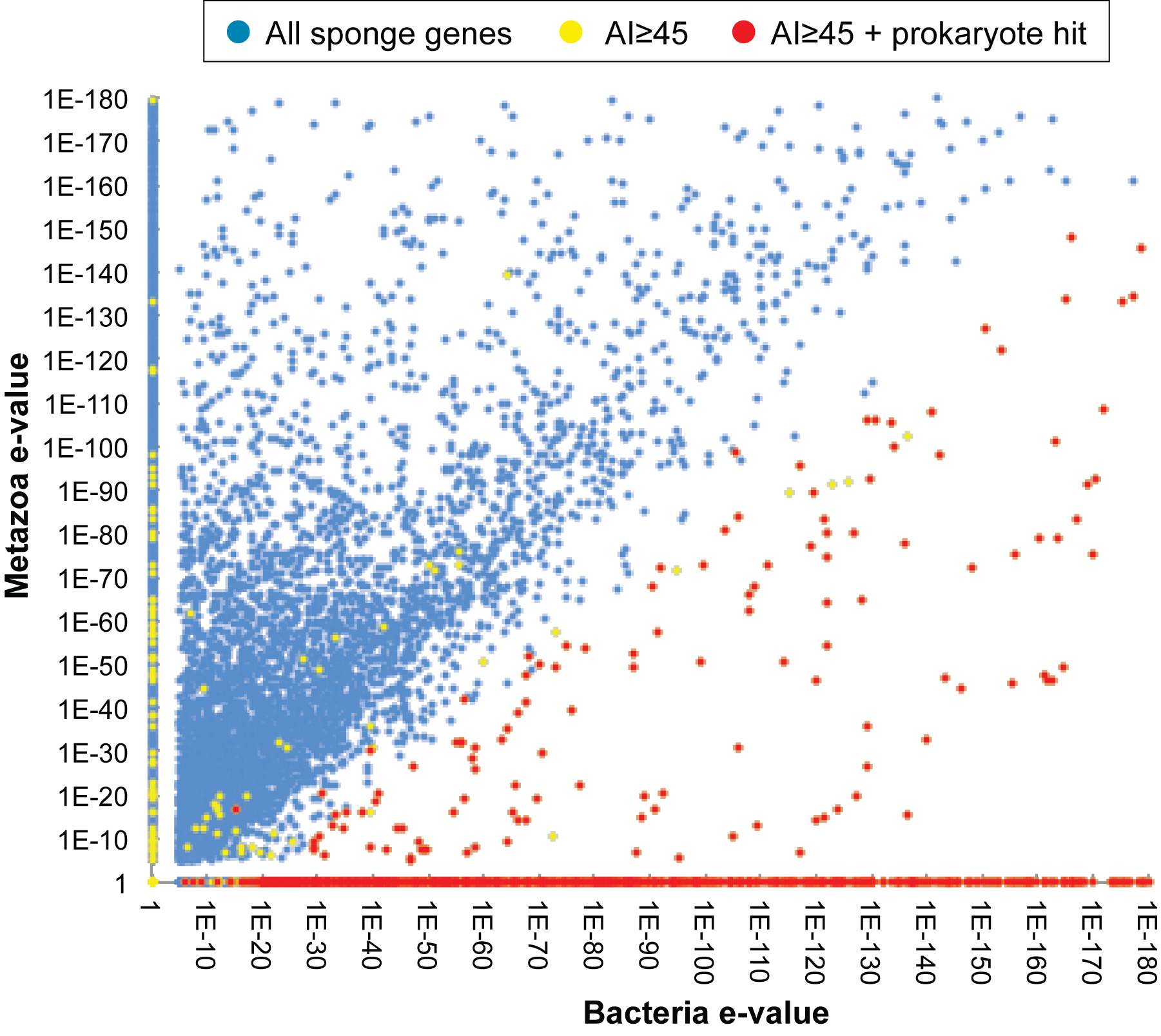

Supplement: S3 Fig — Comparison of the e-values for the best metazoan hit and best bacteria hit for sponge genes reveals that those with AI≥45 are affiliated more closely with the bacteria axis. To further enrich for potential transfers from prokaryote donors, only genes with AI≥45 and a best Blast hit to a prokaryotic sequence were selected for further analysis (blue, all sponge genes; yellow, AI≥45; red, AI≥45 and a best Blast hit to a prokaryotic sequence). (TIF) [file pone.0151092.s003.tif]

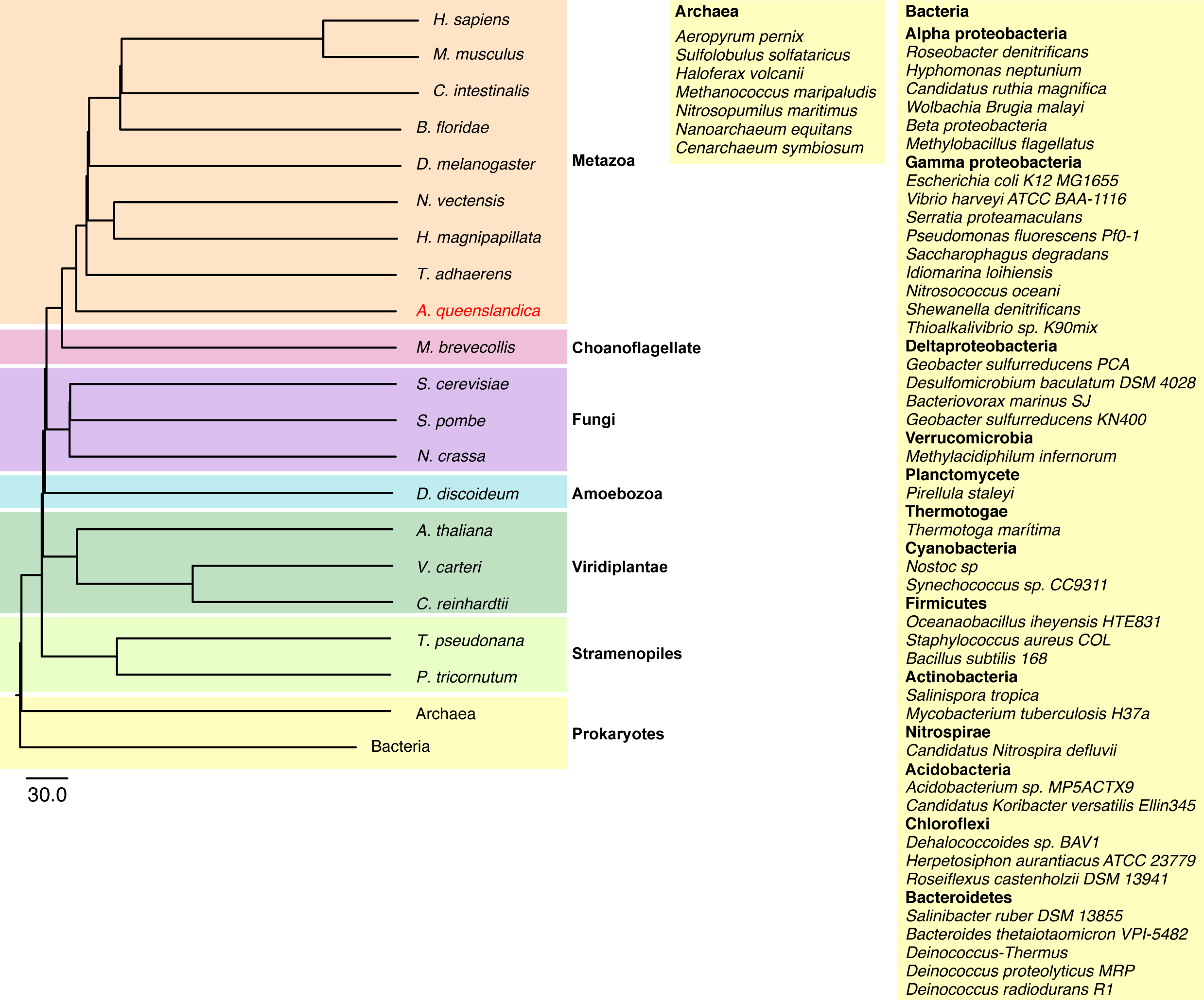

Supplement: S4 Fig — Branch lengths represent average ortholog divergence as computed by EvolMAP. The representative eukaryotic and prokaryotic species included in the analysis are shown. (TIF) [file pone.0151092.s004.tif]
